# Sperm development requires TMC5-dependent mechanoresponsive calcium entry

**DOI:** 10.64898/2026.09.26.754653

**Published:** 2026-09-28

**Authors:** W. Sharon Zheng, Lorena Roa-De-La-Cruz, John Duncan, Liam Slocomb, Betta Chopard, Michelina Kierzek, Fan Lin, Alexander Webb, Erin Jeffery, Celia M. Santi, Jung-Bum Shin, Sameer S. Bajikar, Gloria Shenynkman, Bimal N. Desai, Brian P. Hermann, Christopher B. Geyer, Seham Ebrahim

## Abstract

Spermiogenesis, the transformation of round spermatids into streamlined, fertilization-competent sperm, represents cellular morphogenesis at its most extreme ^1,2^ . Despite decades of effort, spermiogenesis remains difficult to recapitulate outside the testis ^2,3^ , highlighting the importance of cues within the native tissue environment. The nature of these cues, however, remains poorly understood. Here, we discover that mechanical stimulation elicits Ca ^2+^ entry in spermatids and identify transmembrane channel-like protein 5 (TMC5) as a principal mediator of this response. In both mouse and human testes, TMC5 is selectively expressed in spermatids and localized to the plasma membrane. TMC5 associates with TMC7 and calcium- and integrin-binding (CIB) proteins, mirroring TMC–CIB mechanotransduction complexes in sensory organs ^4,5^ . Mechanistically, loss of *Tmc5* blocks mechanically evoked Ca ^2+^ entry, leading to disruptions in cytoskeletal remodeling and failure of CREMτ-dependent transcriptional programs required for spermiogenesis ^6^ . Thus, TMC5 is required to couple mechanical cues to sperm development, with direct implications for male infertility.

## Full Text Availability

The license terms selected by the author(s) for this preprint version do not permit archiving in PMC. The full text is available from the preprint server.

